# A Non-Invasive Urinary Bile-Acid Marker for Never-Smoker Lung Cancer

**DOI:** 10.64898/2026.07.27.26359037

**Published:** 2026-07-31

**Authors:** Seyon Chung, Huaitian Liu, Mohammed Khan, Tanvi S. Patel, Burchelle Blackman, Rolf E. Swenson, Sharon R. Pine, Frank J. Gonzalez, Curtis C. Harris, Daxesh P. Patel

## Abstract

**Introduction:**

Lung cancer in never-smokers is a growing, biologically distinct entity lacking non-invasive markers. Established urinary markers—creatine riboside (CR) and N-acetylneuraminic acid (NANA)—report tumor-intrinsic metabolism, not carcinogen processing. We investigated 27-nor-5β-cholestane-3α,7α,12α,24R,25S-pentol glucuronide (CPG), a bile-acid glucuronide linked to aryl-hydrocarbon-receptor (AhR)/CYP xenobiotic metabolism.

**Methods:**

Urinary CPG was quantified by UPLC–tandem mass spectrometry in an exploratory (NCI-Maryland; n=846) and validation (Colorado; n=505) cohort of non-small-cell lung cancer cases and frequency-matched controls. Associations with case status, smoking stratum, survival, and discrimination were assessed, using tumor RNA sequencing (n=83) and gene-set enrichment analysis (GSEA).

**Results:**

Urinary CPG was higher in cases than controls in both cohorts (P<0.0001). In never-smokers, cases exceeded smoking-matched controls (P<0.001 and P<0.0001), indicating elevation independent of tobacco exposure. After mutual adjustment for CR and NANA, CPG remained independently associated with case status (exploratory OR 1.58, 95% CI 1.15–2.16; validation OR 3.92, 95% CI 2.47– 6.29), with a modest gain in discrimination. High CPG identified never-smokers with worse survival in both cohorts (P<0.001 and P=0.04), remaining significant after multivariable adjustment only in the exploratory cohort. GSEA showed AhR/CYP xenobiotic and Nrf2 oxidative-stress enrichment in high-CPG tumors; the CPG aglycone carried disease-specific 24R,25S stereochemistry.

**Conclusions:**

Urinary CPG was associated with NSCLC in two retrospective case–control cohorts, including in a smoking-matched never-smoker comparison. High CPG also identified never-smokers with worse survival, remaining independently prognostic after adjustment in the exploratory cohort. Tumor expression does not establish tissue of origin. Prospective validation against CR and NANA is required.

## Full Text Availability

The license terms selected by the author(s) for this preprint version do not permit archiving in PMC. The full text is available from the preprint server.

